# Mechanistic Insights Into Inflammation-Induced Arrhythmias: A Simulation Study

**DOI:** 10.3389/fphys.2022.843292

**Published:** 2022-05-30

**Authors:** Xiangpeng Bi, Shugang Zhang, Huasen Jiang, Wenjian Ma, Yuanfei Li, Weigang Lu, Fei Yang, Zhiqiang Wei

**Affiliations:** ^1^ College of Computer Science and Technology, Ocean University of China, Qingdao, China; ^2^ Department of Educational Technology, Ocean University of China, Qingdao, China; ^3^ School of Mechanical, Electrical and Information Engineering, Shandong University, Weihai, China

**Keywords:** inflammation, COVID-19, cardiac simulation, ventricular arrhythmia, rat ventricle

## Abstract

Cardiovascular diseases are the primary cause of death of humans, and among these, ventricular arrhythmias are the most common cause of death. There is plausible evidence implicating inflammation in the etiology of ventricular fibrillation (VF). In the case of systemic inflammation caused by an overactive immune response, the induced inflammatory cytokines directly affect the function of ion channels in cardiomyocytes, leading to a prolonged action potential duration (APD). However, the mechanistic links between inflammatory cytokine-induced molecular and cellular influences and inflammation-associated ventricular arrhythmias need to be elucidated. The present study aimed to determine the potential impact of systemic inflammation on ventricular electrophysiology by means of multiscale virtual heart models. The experimental data on the ionic current of three major cytokines [i.e., tumor necrosis factor-α (TNF-α), interleukin-1 (IL-1β), and interleukin-6 (IL-6)] were incorporated into the cell model, and the effects of each cytokine and their combined effect on the cell action potential (AP) were evaluated. Moreover, the integral effect of these cytokines on the conduction of excitation waves was also investigated in a tissue model. The simulation results suggested that inflammatory cytokines significantly prolonged APD, enhanced the transmural and regional repolarization heterogeneities that predispose to arrhythmias, and reduced the adaptability of ventricular tissue to fast heart rates. In addition, simulated pseudo-ECGs showed a prolonged QT interval—a manifestation consistent with clinical observations. In summary, the present study provides new insights into ventricular arrhythmias associated with inflammation.

## 1 Introduction

Inflammation is a part of the complex biological response of body tissues to harmful stimuli, such as pathogens, damaged cells, toxic compounds, or irradiation (Chen et al., 2018). These harmful stimuli trigger a cascade that releases inflammatory biomarkers and recruits immune cells, which contribute to eliminating the initial cause of cell injury and initiating tissue repair. However, an excessive immune response could potentially lead to multiorgan dysfunction by triggering a cytokine storm.

According to World Health Organization statistics, cardiovascular diseases (CVDs) are the number one cause of death globally, accounting for an estimated 17.9 million lives each year (Kaptoge et al., 2019). In some recent studies, accumulating data suggest increased CVD morbidity and mortality in patients infected with coronavirus disease 2019 (COVID-19), among which there may be an arrhythmia effect (Lazzerini et al., 2020a, 2020b; O’Shea et al., 2021). The mechanisms underlying COVID-19-related arrhythmia events are complicated. For example, CVDs in these patients can be caused by immune cell tissue invasion associated with pulmonary or cardiogenic myocardial injury (Agricola et al., 2020; Jaffe et al., 2020; Magadum and Kishore, 2020). Recently, clinical research by Lazzerini et al. reported that the QT interval was prolonged in patients with COVID-19, and this electrocardiogram (ECG) abnormality was accompanied by high levels of inflammatory cytokines in serum, suggesting a potential link between systematic inflammation and cardiac arrhythmias (Lazzerini et al., 2020a).

There is increasing experimental evidence supporting the effects of inflammatory cytokines (mainly tumor necrosis factor-α (TNF-α), interleukin-1β (IL-1β), and interleukin-6 (IL-6)) on cardiac ion channels, and this specific type of channelopathy is termed *inflammatory cardiac channelopathy* (Lazzerini et al., 2018, 2019). Existing studies have found that inflammatory cytokines can affect multiple ion channels, including transient outward potassium current (*I*_to_) (Kawada et al., 2006; Fernández-Velasco et al., 2007; Grandy and Fiset, 2009; Monnerat et al., 2016), rapid delayed-rectifier potassium channel (*I*_Kr_) (Wang et al., 2004; Aromolaran et al., 2018), and L-type calcium current (Hagiwara et al., 2007). There are also studies suggesting the effects of inflammatory cytokines on calcium handling. For example, IL-6 was reported to inhibit the gene expression of sarco/endoplasmic reticulum Ca^2+^-ATPase (SERCA) (Villegas et al., 2000; Tanaka et al., 2004), and IL-1β was observed to increase sarcoplasmic reticulum (SR) calcium leakage (Monnerat et al., 2016). Although the effects of inflammatory cytokines on individual ion channels have been investigated in these studies, its integral effect on ventricular cellular action potential (AP) and its conduction properties remain unclear. In recent years, emerging cardiac simulations have provided powerful tools for exploring the pathogenesis of cardiovascular diseases (Xie et al., 2004; Arevalo et al., 2016; Zhang et al., 2019, 2020). In our recent work, we constructed a multiscale ventricle model that is able to reproduce both physiological and pathological phenomena on different scales (Bi et al., 2021). Based on this multiscale model, we investigated and evaluated the effects of inflammatory cytokines on ventricular electrophysiology.

The present study aimed to determine the potential impact of systemic inflammation on ventricular electrophysiology. Several simulations were conducted in this work. First, available experimental data regarding the effects of several inflammatory cytokines on multiple cardiac targets were incorporated into rat and human ventricular myocyte models so that the inflammation-induced electrophysiological alterations at the cellular level could be simulated. Next, we constructed a 1-D strand model and quantitatively evaluated the temporal susceptibility of inflammatory tissue to unidirectional conduction blocks. Finally, inflammatory cells were coupled to form a local inflammatory area, which was then incorporated into a ventricular slice model to explore the potential proarrhythmic factors under local inflammatory conditions. As a parallel experiment, we also simulated the electrical activities under global inflammatory conditions by setting all of the cells on the slice as inflammatory cells.

## 2 Methods

### 2.1 Effects of Inflammatory Cytokines

Evidence from several *in vitro* and animal studies indicated that an overactive immune response might lead to a storm of inflammatory cytokines, and some of these inflammatory cytokines directly affect the function of ion channels in cardiomyocytes. In this research, we mainly evaluated the effects of three cytokines (i.e., TNF-α, IL-1β, and IL-6) on cardiomyocytes and their possible proarrhythmic effects. It has been demonstrated that these cytokines can prolong the ventricular action potential duration (APD) by modulating several targets in cardiomyocytes, specifically the transient outward K^+^ channel (*I*_to_), the rapid delayed-rectifier K^+^ current (*I*_Kr_), and some targets involved in calcium handling. Focusing on *acute* inflammation, we screened out the experimental data based on the duration of the experimental treatment (less than 48 h), which are listed in Table 1, Table 2, and Table 3.

**TABLE 1:** The effects of TNF-α on cellular targets.

Targets	Experimental observations				References
	Effects	Time of treatment	Concentration	Type of cell tested	—
*I*_to_	Current density: −23.4–65% Inactivation curve: Approximately 5.7 mV shift to the left	48 h	1–5 ng/ml	Ventricular myocyte (rat)	Fernández-Velasco et al. (2007)
*I*_Kr_	Current density: −33%	10 h	1 ng/ml	HEK293	Wang et al. (2004)

**TABLE 2:** The effects of IL-6 on cellular targets.

Targets	Experimental observations				References
	Effects	Time of treatment	Concentration	Type of cell tested	—
*I*_Kr_	Current density: −29.6% Activation curve: 5 mV shift to the left	40 min	20 ng/ml	HEK293	Aromolaran et al. (2018)
*I*_CaL_	Current density: +27%	30 min	20 ng/ml	Ventricular myocyte (mice)	Hagiwara et al. (2007)
J_up_	Expression of SERCA gene: −21%∼−50%	48 h	10 ng/ml	Ventricular myocyte (rat)	Villegas et al. (2000)

**TABLE 3:** The effects of IL-1β on cellular targets.

Targets	Experimental observations				References
	Effects	Time of treatment	Concentration	Type of cell tested	—
*I*_to_	Current density: −36.8%	24 h	60 pg/ml	Ventricular myocyte (rat)	Monnerat et al. (2016)
J_leak_	SR Ca^2+^ leak:+63.6%	24 h	60 pg/ml	Ventricular myocyte (rat)	Monnerat et al. (2016)

### 2.2 Single-Cell Simulations

The rat ventricular cell model by Terkildsen et al. (referred to as the Terk model) (Terkildsen et al., 2008) and the human ventricular cell model by Ten Tusscher et al. (referred to as the TP06 model) (Ten Tusscher et al., 2006) were adopted in this study. Due to the lack of heterogeneity in the Terk model, AP heterogeneities, including transmural heterogeneity and interventricular heterogeneity, were incorporated according to our previous study (Bi et al., 2021) and experimental observations (Clark et al., 1993; Shimoni et al., 1995; Casis et al., 1998; MacDonell et al., 1998; Kaprielian et al., 1999; Ashamalla et al., 2001).

In the single-cell simulation, the rat model was paced with a series of 1000 stimuli with an amplitude of 6 pA/pF and a duration of 5.0 ms (80 pA/PF, 0.5 ms in the TP06 model) to reach the steady-state. To investigate the effects of a single cytokine and the combined influences of multiple cytokines on the cardiomyocytes, we adjusted the conductance of the related channel or ion flux of the related calcium handling process in the cell models according to the previous experimental recordings (Table 1, Table 2, Table 3). Note that the combined effects of the three cytokines were assumed to be an accumulation of each cytokine. The AP traces, APD_90_, and current density traces of the different types of cells under various conditions were recorded for later analysis.

In addition, the data used in this study were obtained from bioexperiments in which the preparation concentrations of the cytokines were higher than the clinically measured cytokine levels in patients (Liu and Zhao, 1999; Monnerat et al., 2016; Liu et al., 2021). Therefore, we also considered another ‘mild’ type of inflammation (referred to as *mild inflammation* in this study) by halving the reported effects of cytokines as shown in Table 1, Table 2, Table 3.

### 2.3 One-Dimensional (1-D) Simulations Using Transmural Tissue Strand Models

#### 2.3.1 Numerical Details

A 15-mm-long 1-D transmural tissue strand model of humans was constructed using the monodomain equation:

$$\frac{\partial V_{m}}{\partial t}=\nabla \cdot \text{D}\nabla V_{m}-\frac{I_{ion}}{C_{m}}$$ (1)

where $V_{m}$ is the membrane voltage, $I_{ion}$ is the sum of the currents that flow through the membrane, and $C_{m}$ is the membrane capacitance. The 1-D model was discretized by a spatial resolution of 0.15 mm to form 100 interconnected nodes. The proportions for the transmural cell types were set to 25:35:40 for endocardial (ENDO), middle (MID), and epicardial (EPI) cells to produce a positive going T-wave, in accordance with our previous work (Jiang et al., 2022). The diffusion coefficient D was set to 0.154 mm^2^/ms, and the corresponding conduction velocity (CV) was 0.74 m/s through the strand. In addition, there is evidence suggesting that cell-to-cell coupling in tissue is reduced under inflammation (Baum et al., 2012). Therefore, the conduction coefficient in the inflammatory area was set to 0.1 mm^2^/ms (CV: 0.6 m/s) to simulate cell coupling under inflammation.

#### 2.3.2 Measurements of the Vulnerable Window

A *vulnerable window* (VW) is a certain time period in which a unidirectional conduction block occurs. A standard S1–S2 protocol was used to measure the VWs across the whole tissue strand. Specifically, a series of supra-threshold stimuli (S1) were applied to the first three cells at the ENDO end with a frequency of 1 Hz. After an interval (Δt), a premature stimulus was applied to a 0.45 mm segment centered on the location currently being measured. Due to the different refractory durations, different Δt would correspond to different results: bidirectional conduction block, unidirectional conduction block, and bidirectional conduction. The width of the VWs across the strand was averaged by the cell number, which acted as a metric for the temporal vulnerability to arrhythmias.

### 2.4 Two-Dimensional (2-D) Simulations Using Realistic Ventricular Slice Models

#### 2.4.1 Model Geometries and Numerical Details

Two geometries of 2-D realistic ventricular tissue for rats and humans were employed in this study. Preprocessing of geometries, including transmural layer segmentation, was conducted according to our previous work (Bi et al., 2021). The proportions of transmural layers in humans were consistent with the aforementioned transmural settings in the 1-D strand model and were 2:1 for ENDO:EPI in rats.

Similar to the 1-D model, the monodomain equation (Eq. 1) was adopted to describe the propagation of excitation waves in the ventricular slice. Isotropic propagation was assumed, and the diffusion coefficient D was set to 0.08 mm^2^/ms and 0.154 mm^2^/ms in rats and humans, respectively, to produce CVs of 0.42 m/s for rats (Sedmera et al., 2016) and 0.74 m/s for humans (Taggart et al., 2000). It should be noted that there is evidence suggesting that cell-to-cell coupling is reduced by 30–55% in inflammatory tissue (Baum et al., 2012); therefore, the conductivity coefficient was reduced by 35% in the model for inflammation to reflect this reduction. The spatial step was set to 0.1 mm in rats and 0.15 mm in humans to be consistent with the reported cell length (i.e., 80–150 μm (Hinrichs et al., 2011)). To mimic the physiological characteristics of the Purkinje fibers, a series of supra-threshold stimuli were applied to several pacing sites on the endocardium of the slice.

#### 2.4.2 Model Settings for the Inflammatory Conditions

Two inflammatory conditions, namely, local inflammation and global inflammation, were discussed in this study. Specifically, cells that incorporated the effects of the inflammatory cytokines were regarded as ‘inflammatory cells’. In the local inflammatory condition, a group of normal cells within a local area on the free wall of the left ventricle were replaced by inflammatory cells (Supplementary Figure S1), whereas all cells were set as inflammatory cells under the global inflammatory condition.

#### 2.4.3 Initiation of Reentry Arrhythmias in 2-D Ventricular Slices

A typical S1–S2 protocol (Sutanto et al., 2020; Cluitmans et al., 2021) was used to induce reentry arrhythmias in 2-D slice models. Specifically, under physiological conditions, the premature S2 stimulus was applied to a local region of the epicardium within the VW caused by transmural repolarization heterogeneity. In contrast, due to the presence of pathological heterogeneity in the local inflammatory condition, S2 was applied to the boundary between the normal and inflammatory areas. The above process of applying S2 stimulation may be repeated several times until S2 falls within the VW, thus producing a unidirectional conduction block.

#### 2.4.4 Measurements of the Critical Pacing Cycle Length

The critical pacing cycle length (PCL) was defined as the minimum pacing cycle length for maintaining a normal 1:1 conduction in 2-D ventricular slices. In this study, we tested the critical PCL under control and global inflammatory conditions. For both cases, we gradually decreased the PCL until reaching a critical value under which the tissue failed to maintain a normal 1:1 conduction.

#### 2.4.5 Generation of the Pseudo-ECG

The pseudo-ECG was calculated from the 2-D ventricular slice by the following equation:

$$\phi \left(x^{\prime },y^{\prime }\right)=\frac{a^{2}\sigma_{i}}{4\sigma_{e}}\int \left(-\nabla V_{m}\right)\cdot \left[\nabla \frac{1}{r}\right]d\Omega$$ (2)

where $V_{m}$ is the membrane potential, ϕ is a unipolar potential generated by the tissue, r is the distance between a source point and the virtual electrode, $\sigma_{i}$ and $\sigma_{e}$ are the extracellular and intracellular conductivities, respectively, and ∫ is the domain of integration. The models were paced to their steady states at 1 Hz before being used to calculate ECGs.

## 3 Results

### 3.1 Effects of Inflammatory Cytokines at the Cellular Level

**FIGURE 1:**
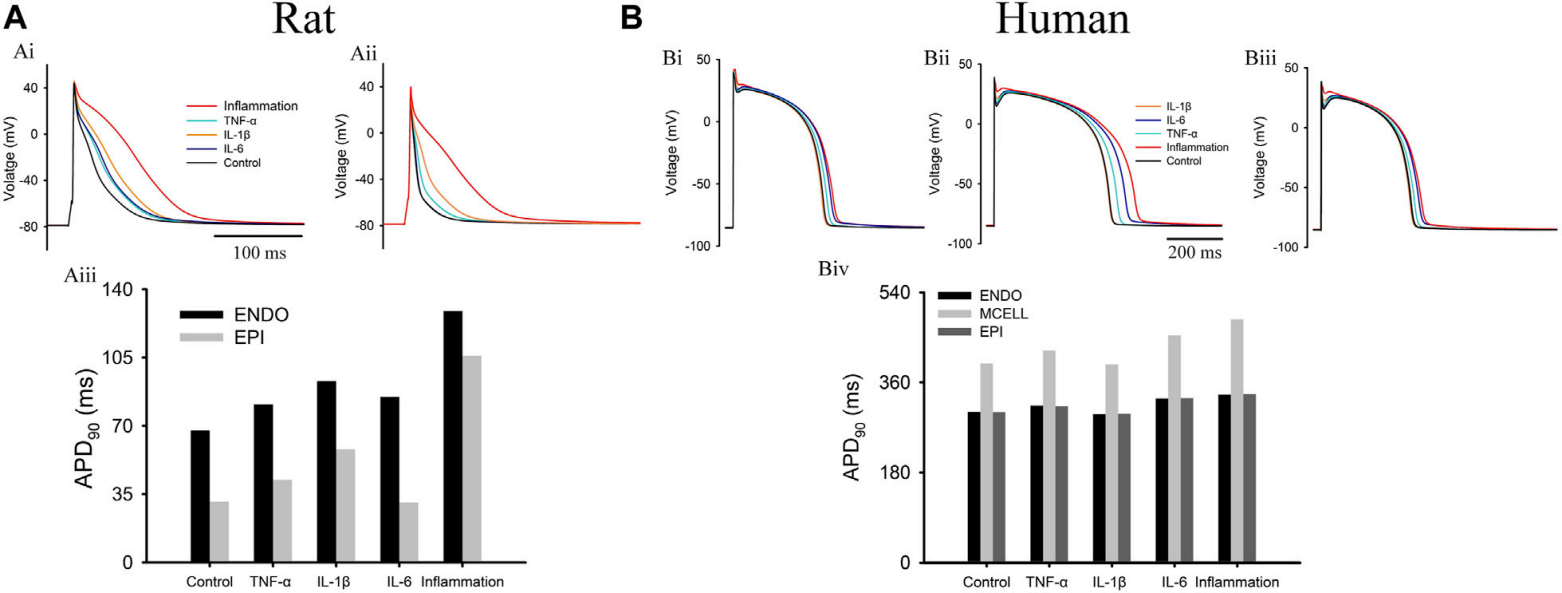
Effects of different inflammatory cytokines on action potentials in **(A)** rat and **(B)** human models. Top panels plot the APs in **(Ai)** rat ENDO **(Aii)** rat EPI, **(Bi)** human ENDO, **(Bii)** human MID, and **(Biii)** human EPI cells. The bottom panels are the APD_90_ of different cells for **(Aiii)** rats and **(Biv)** humans. Note: “inflammation” represents the combined effects of three cytokines.

The individual effect of each inflammatory cytokine and their combined effects (called “inflammation” in this study) on APs (1 Hz) are shown in Figure 1. First, for the rat cell model, it can be observed that except for IL-6 causing a negligible influence on EPI AP, all of these cytokines caused obvious AP prolongations in the ENDO/EPI cells. In terms of APD_90_, both EPI and ENDO cells exhibited a significant increase compared with their control levels, which hinted at the presence of severe pathological repolarization heterogeneity between normal and inflammatory tissue. Next, in the human cell model, the simulation results (Figure 1B) showed that the APs in the IL-6 and TNF-α groups were prolonged in all three types of cells, but there was little change in AP in the IL-1β group. Moreover, as Figure 1Biv shows, the ΔAPD between MID and ENDO/EPI exhibited an obvious augmentation (from 97 to 150 ms) under inflammatory conditions, leading to a larger transmural repolarization heterogeneity compared with the control level.

**FIGURE 2:**
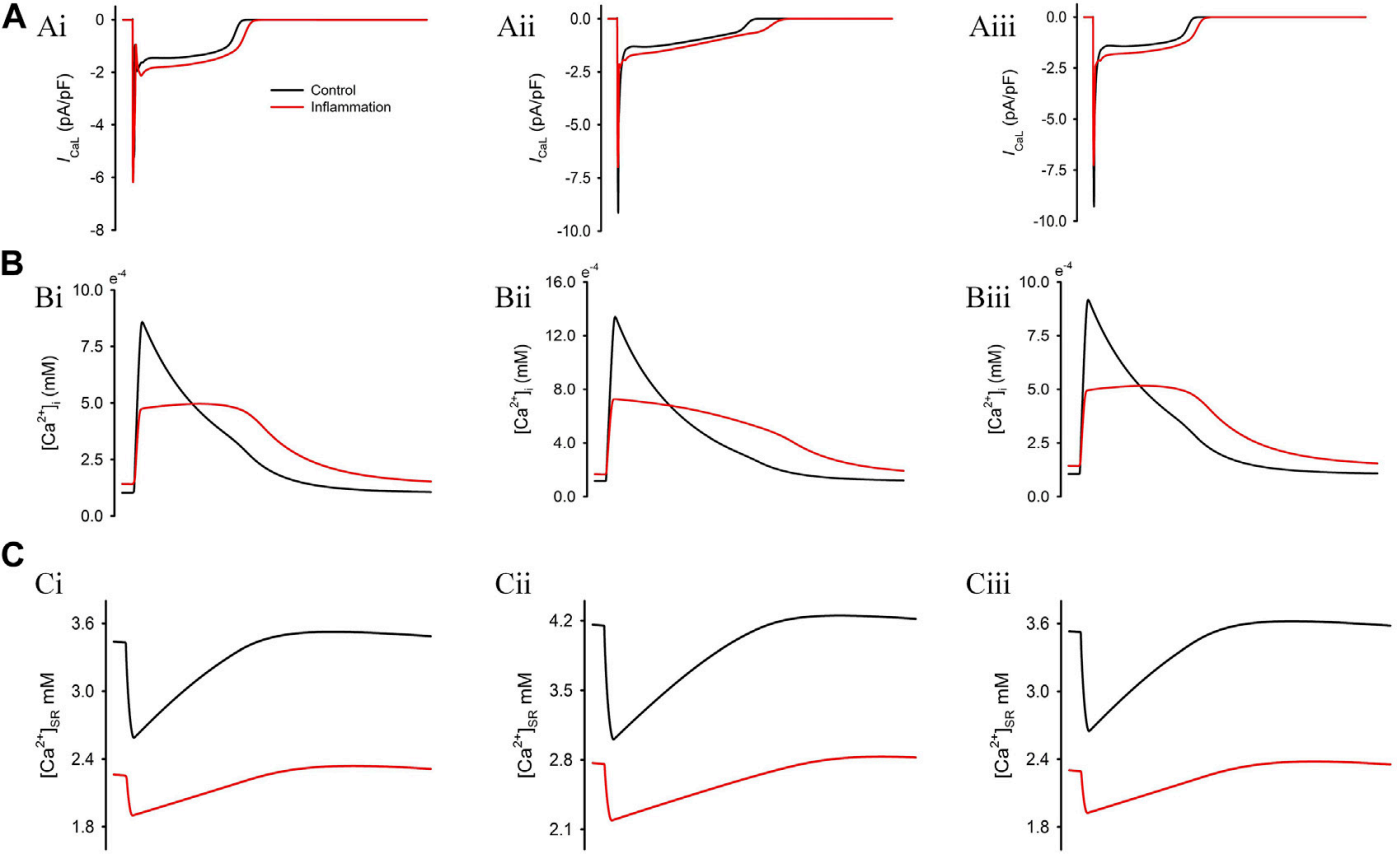
Simulation results of the combined effects of three inflammatory cytokines on calcium handling. Corresponding profiles for *I*_CaL_ **(A)** and the concentration of Ca^2+^ in the cytosol **(B)** and sarcoplasmic reticulum **(C)**.

In addition to the influences on AP, we also investigated the alteration of calcium handling using the TP06 model, as shown in Figure 2. In this regard, the reported reduced systolic [Ca^2+^]_i_ (Sugishita et al., 1999) and elevated diastolic [Ca^2+^]_i_ (London et al., 2003) under inflammatory conditions were successfully reproduced (Figure 2B). This observation might be attributed to the reduced SERCA activity and the increased SR Ca^2+^ leakage, which also caused a decreased SR Ca^2+^ content (Figure 2C). Moreover, the greatly decreased peak Ca^2+^ concentration in the cytoplasm exactly reflected the negative inotropic effect (Weisensee et al., 1993; Sugishita et al., 1999; Duncan et al., 2010) under inflammatory conditions.

**FIGURE 3:**
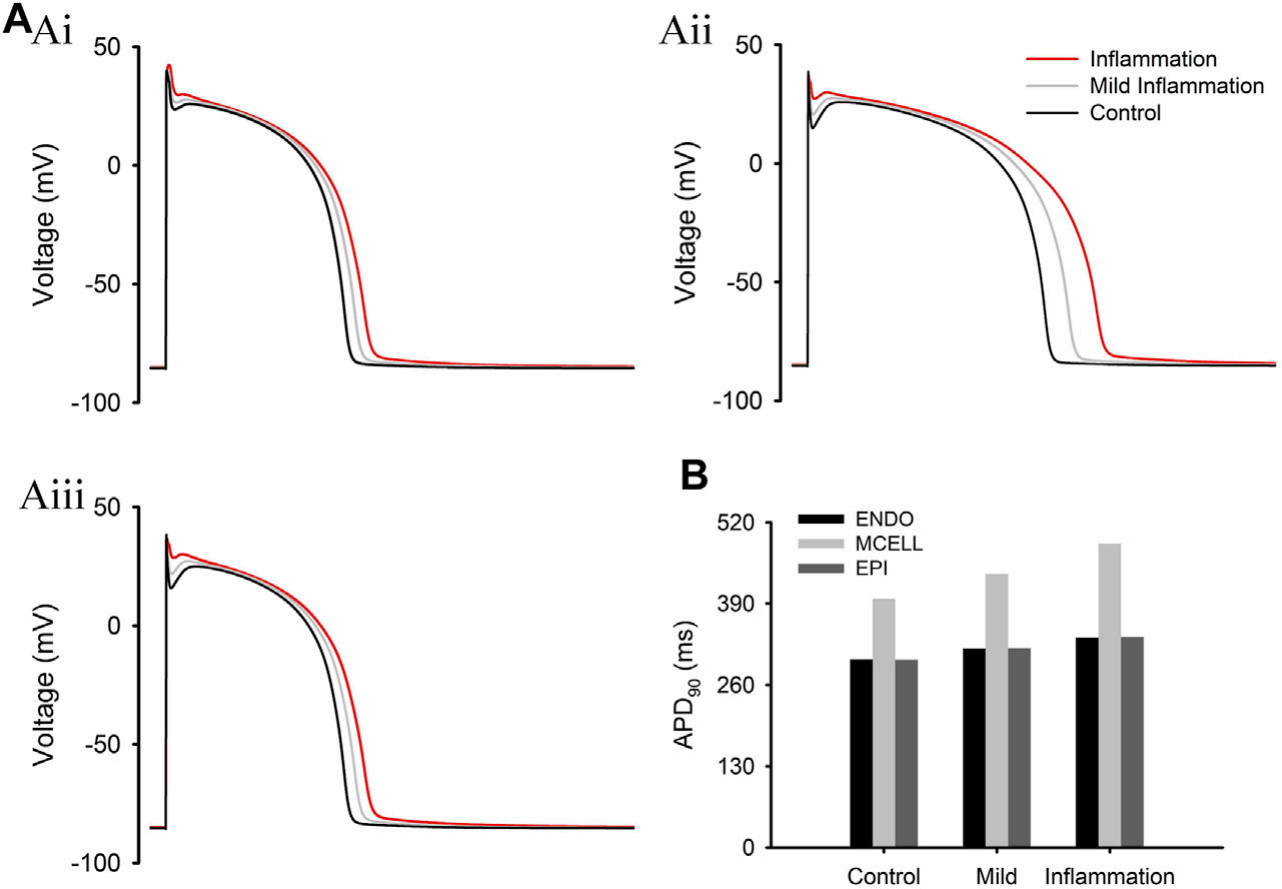
Simulation results under different levels of inflammation. **(A)** APs of ENDO **(Ai)**, MID **(Aii)**, and EPI **(Aiii)** cells at different levels of inflammation. **(B)** APD_90_ of three types of cells under normal and inflammatory conditions.

The above simulation results were based on experimental data using high doses of cytokines. In this study, we also tested a type of *mild* inflammation by downregulating the reported effects in the TP06 model. The simulation results are shown in Figure 3. We can see that APD varied with the degree of inflammation, and in the case of mild inflammation, the ΔAPD between MID and ENDO/EPI cells showed a slight increase (from 97 to 119 ms) compared with the control group.

The other ionic current traces in the rat and human cell models under different cytokines can be found in Supplementary Figures S2,S3.

### 3.2 Evaluation of the Temporal Vulnerability to Unidirectional Conduction Blocks Under Inflammatory Conditions

**FIGURE 4:**
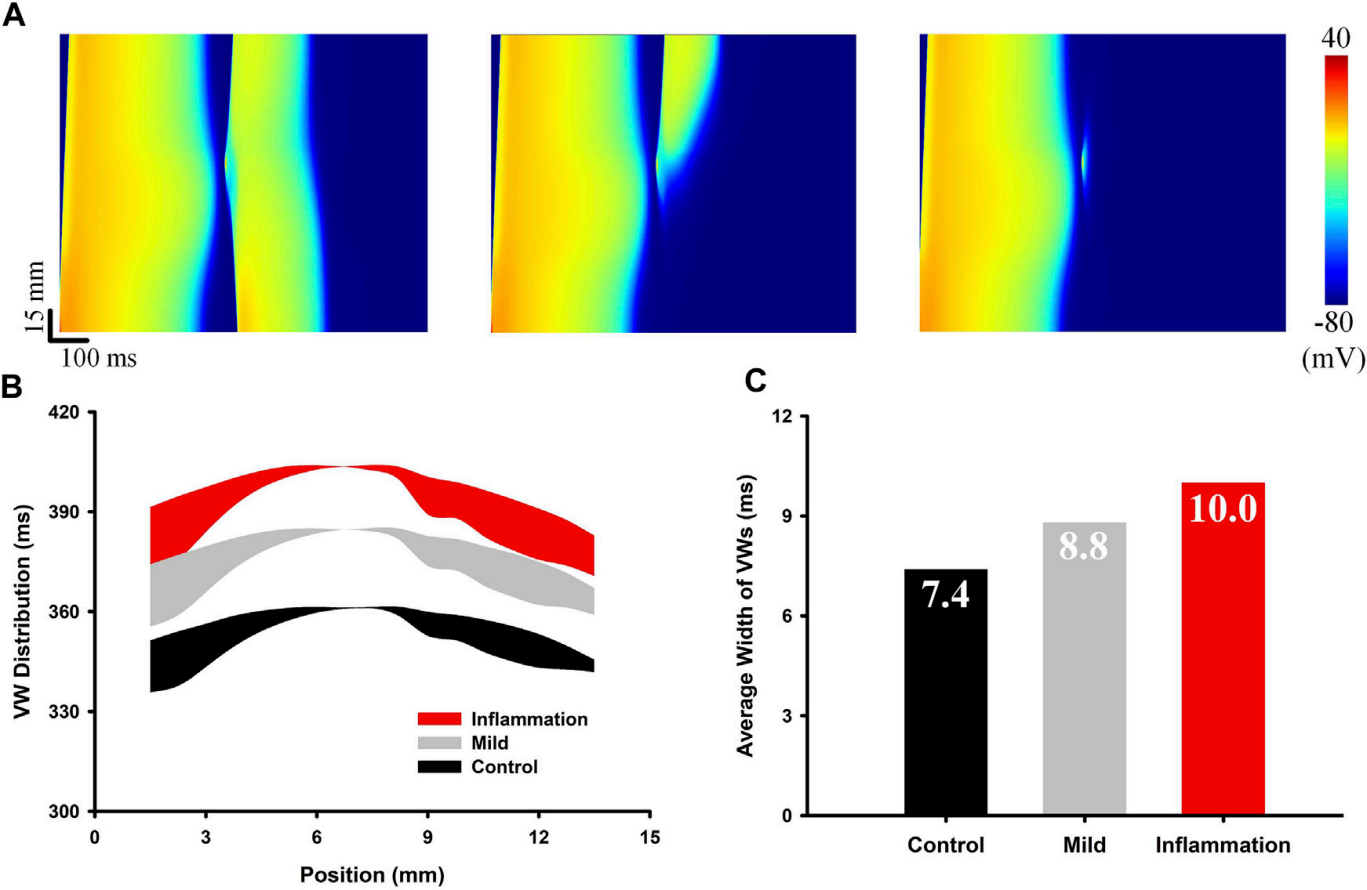
Measurement of the VW in the 1-D strand model under normal, mild inflammatory, and extreme inflammatory conditions. **(A)** The three subplots from left to right show the bidirectional conduction, the unidirectional conduction block, and the bidirectional conduction block. **(B)** Distributions of VWs across the strand. Black and red belts represent the control and inflammatory conditions, respectively. **(C)** Comparison of the average width of the VWs in the three groups.

Unidirectional conduction blocks are an important pathological phenomenon in 1-D tissue, as the unidirectionally propagated excitation wave can evolve into reentrant spiral/scroll waves in 2-D slices and 3-D organs. The time window within which unidirectional conduction blocks occur, termed the *vulnerable window*, is a commonly used metric for measuring the temporal susceptibility of tissue to arrhythmias. In this section, we quantified the influence of inflammation on temporal vulnerability by measuring the VW across the 1-D transmural strand. Different degrees of inflammation, i.e., an extreme level of inflammation (e.g., sepsis) and a mild level of inflammation, were evaluated individually. The simulation results are shown in Figure 4. Specifically, Figure 4A shows different responses to the premature S2 stimulus, including bidirectional conduction (S2 too early), unidirectional conduction block (VW), and bidirectional conduction block (S2 too late). The distribution of the time window when unidirectional conduction blocks occurred (i.e., the distribution of VW) is plotted in Figure 4B. Both mild inflammation and inflammation delayed the occurrence of VW, and the average width of the VWs increased gradually from 7.4 to 8.8 and 10.0 ms depending on the degree of inflammation. As a wider VW signifies a higher chance of a unidirectional conduction block, the above simulation results implied an increased temporal susceptibility to reentry arrhythmias under inflammatory conditions.

### 3.3 Evaluation of the Proarrhythmic Effects of Pathological Heterogeneity Under Local Inflammatory Conditions

**FIGURE 5:**
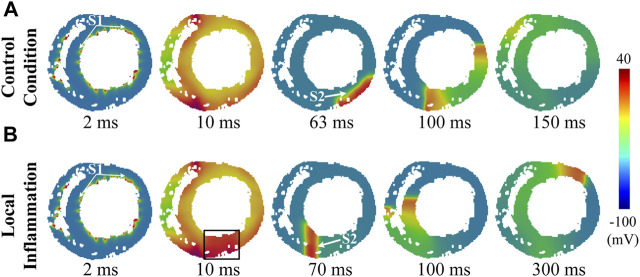
Induced reentry arrhythmias in rat ventricular slices under conditions of **(A)** control and **(B)** local inflammation. The S1 and S2 stimuli are marked by white arrows, while the inflammatory region is indicated by a black rectangle.

**FIGURE 6:**
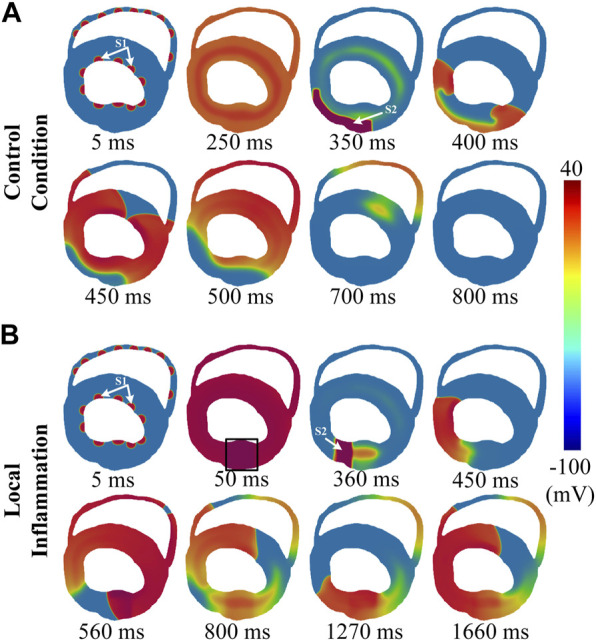
Induced reentry arrhythmias in rat ventricular slices under conditions of **(A)** control and **(B)** local inflammation. The S1 and S2 stimuli are marked by white arrows, while the inflammatory region is indicated by a black rectangle.

Inflammation may initially occur in a specific area in the heart. This type of inflammation, termed *local inflammation* in this study, may result in the presence of pathological heterogeneity in ventricular tissue due to the prolonged APD of the affected cells, which predisposes to ventricular arrhythmias. In this section, we mimicked this condition by setting a local region of inflammation on the free wall of the left ventricle, and a typical S1-S2 protocol was used to evaluate the inducibility of reentry arrhythmias in this condition (see *Methods* for more details). The simulation results are shown in Figure 5 (rat) and Figure 6 (human).

First, for the control condition (Supplementary Video S1), it can be observed that, due to the limited size of rat hearts, the evoked unidirectional conduction was not able to turn back to form a functional reentry, and the subsequent two waves propagating along the ventricular wall collided and failed to form anatomical reentry (snapshots in Figure 5A). The whole process lasted only approximately 90 ms. For the human ventricular slice (Supplementary Video S2), although the evoked unidirectional conduction could turn back to form a functional reentry, such a process was unsustainable, and the spiral waves terminated shortly after the first cycle with a brief lifetime of approximately 350 ms (snapshots in Figure 6A).

Simulation results of local inflammatory conditions are shown in Figure 5B (rat) and Figure 6B (human). Due to the asynchronous repolarization caused by the prolonged APD of inflammatory cells, extra S2 stimulation applied to the border area would encounter the refractory tail on the inflammatory side. Therefore, S2 would generate a unidirectional conduction block, which in turn would evolve gradually into an anatomical spiral wave circling around the ventricular ring structure (Supplementary Video S3,S4). In brief, the pathological heterogeneity caused by inflammatory cytokines provided extra substrates for unidirectional conduction block and reentry arrhythmias.

### 3.4 Evaluation of the Adaptability of Tissue to High Stimulating Frequencies Under Global Inflammatory Conditions

**FIGURE 7:**
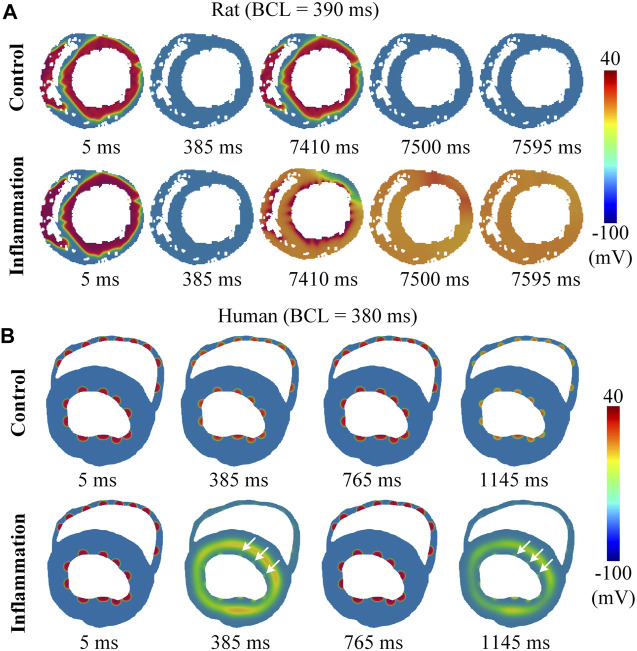
The performance of the model at high frequency. Conduction waves under physiological and inflammatory conditions at high frequency **(A)** for rats and **(B)** for humans. The white arrow represents stimulus S1, which failed to pace.

Another type of inflammatory condition, as opposed to local inflammation, is *global inflammation*. It reflects a globally affected condition in which the whole ventricle is influenced by inflammatory cytokines. Compared to its counterpart, global inflammation does not create additional pathological heterogeneity; however, the prolonged wavelength may impair the tissue’s adaptability to fast heart rates. In this study, we mimicked the global inflammatory condition by setting all cells as inflammatory cells and tested the influences of global inflammation on the critical PCL using rat and human ventricular models (see *Methods* for more details). In the rat model, the measured critical PCL was 101 ms (9.9 Hz) in control conditions, while in global inflammation, it increased to 390 ms (2.56 Hz), and stimuli with cycle lengths below 390 ms led to complete repolarization failure (see Figure 7A or Supplementary Video S5). The critical PCL of 390 ms corresponds to a heart rate of 154 bpm, which is significantly lower than the physiological range of approximately 300–500 bpm in rats. Such simulation results showed a decreased adaptability of tissue to fast heart rates under global inflammation conditions and suggested a strong proarrhythmic effect. However, considering that the heart rate of rats is much faster than that of humans, this result might be species dependent. In addition, the atypical phenomenon of complete repolarization failure might also depend on the specific model (Supplementary Figure S6). To further clarify these questions, we performed parallel simulations using human tissue models. The simulation results showed that complete repolarization failure did not occur even at very high frequencies; however, there was still a critical PCL below which the 1:1 conduction could not be maintained and was replaced by 2:1 conduction (see Figure 7B or Supplementary Video S6). In this setting, the critical PCL was increased from 319 ms in the control condition to 380 ms in the global inflammatory condition. Notably, the BCL of 380 ms, which corresponds to a heart rate of approximately 157 bpm, was physiologically relevant and therefore might lead to arrhythmogenesis.

### 3.5 Pseudo-ECGs in 2-D Simulation

**FIGURE 8:**
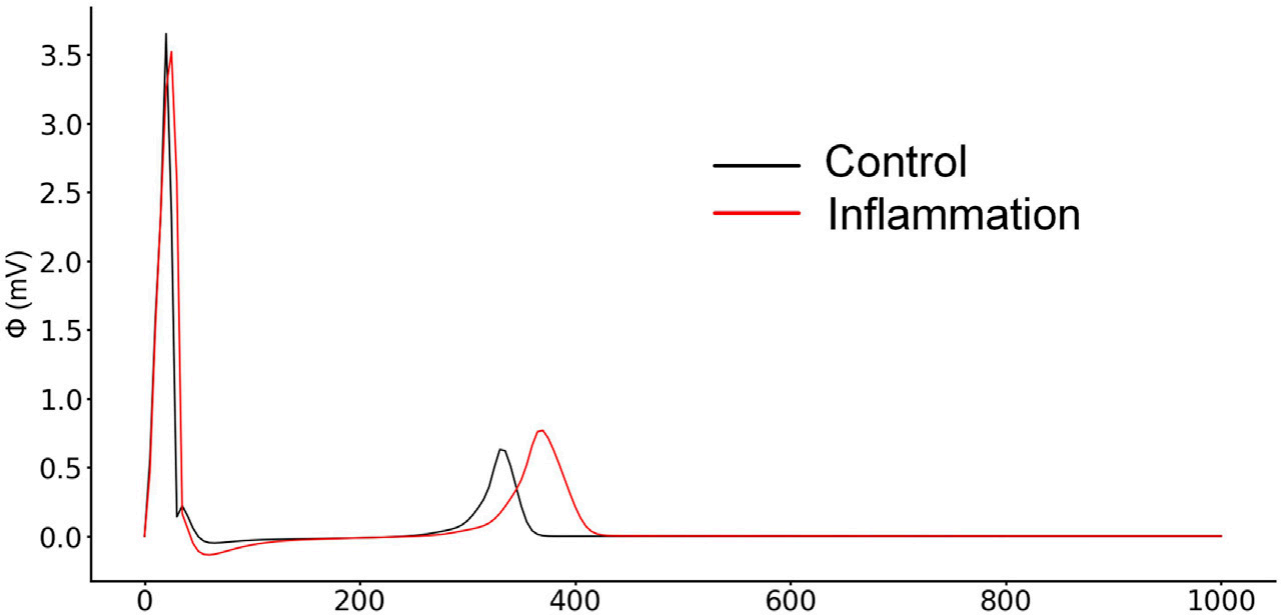
Comparison of pseudo-ECGs between normal and inflammatory conditions.

The generated pseudo-ECG of the human ventricular 2-D slice model under physiological and inflammatory conditions is shown in Figure 8. As some available studies concerning systemic inflammation have reported (Adlan et al., 2015; Lazzerini et al., 2020a; Armbruster et al., 2022), prolonged QT intervals were also reproduced in our simulation results.

## 4 Discussion

### 4.1 Main Findings

In recent years, accumulating evidence has shown an association between systemic inflammation and cardiovascular disease. Inflammatory cytokines, a type of signaling molecule secreted from immune cells, were proven to be able to affect membrane ion channels and might therefore lead to ventricular arrhythmias. In this study, we selected three of these major cytokines and evaluated their proarrhythmic effects using a multiscale virtual heart. The main findings are as follows: 1) at the cellular level, inflammatory cytokines caused a prolongation of APD by affecting multiple ion channels, and heterogeneously prolonged APDs led to augmented transmural heterogeneities; 2) simulation results of the VW using the 1-D strand suggested that inflammation increased the temporal vulnerability to arrhythmias; 3) in the case of local inflammation, the repolarization of the inflammatory area was delayed due to the cytokine-induced APD prolongation, leading to the presence of pathological heterogeneities around the local inflammatory area. Such regional differences in repolarization provided extra substrates for the unidirectional conduction block and increased the chance of the development of anatomical reentry arrhythmias; 4) In the global inflammatory condition, the generated pseudo-ECG exhibited a prolonged QT interval that was in accordance with the clinical observations. Furthermore, the globally prolonged APD impaired the tissue adaptability to high frequencies and caused 2:1 conduction at physiologically relevant heart rates.

APD prolongation has been observed in many pathological conditions and has been shown to be proarrhythmic (Antoniou et al., 2017; Dietrichs et al., 2019; Shimaoka et al., 2020). For example, APD prolongation and changes in electrophysiological characteristics caused by the downregulation of outward potassium currents, alterations of calcium channel kinetics and increases in late sodium currents create a substrate for ventricular arrhythmias in the case of heart failure (Zhang et al., 2018). A study on hypokalemia showed that APD prolongation could predispose patients to early afterdepolarizations, which in turn act as triggers for ventricular arrhythmias (Tse et al., 2021). APD prolongation is also the main factor responsible for the proarrhythmic influences of drugs with cardiotoxicity. A prolonged APD and, consequently, a prolonged QT interval, are considered important biomarkers of drug cardiotoxicity in drug discovery (Schrickel et al., 2006; Hondeghem et al., 2011). For example, the antihistamine drug terfenadine, which was previously used for the treatment of allergic conditions, was proven to be able to prolong APD due to its effects of blocking hERG currents (*I*_Kr_), causing QT prolongation and torsade de pointes (TdP). Despite the tight association between APD/QT prolongation and ventricular arrhythmia, prolonged QT does not always lead to arrhythmogenesis events. Some new evaluation criteria, such as TRIaD (Triangulation, Reverse use dependence, Instability and Dispersion), have been suggested to identify false-positive cases. On the other hand, it should also be noted here that APD prolongation can also exert antiarrhythmic effects by extending the effective refractory period, which is also the major pharmaceutical mechanism of class III antiarrhythmic drugs. In particular, homogeneous APD prolongation in the absence of early afterdepolarizations (EADs) commonly exerts antiarrhythmic effects, whereas EADs or repolarization heterogeneities induced by inhibition of repolarization are proarrhythmic. For the case in this study, the simulation results demonstrated a double-sided nature, especially in a condition of global inflammation: the prolonged refractory period in ventricles tends to be antiarrhythmic, but it also reduces the adaptability of the tissue to high heart rates. For the local inflammatory condition, although we did not observe EADs, the presence of both pathological heterogeneities and augmented transmural heterogeneity increased the vulnerability to arrhythmogenesis in terms of both spatial and temporal aspects.

Accumulating studies have demonstrated that augmented electrophysiological heterogeneity provides proarrhythmic substrates (Laurita and Rosenbaum, 2000; Pak et al., 2004; Antzelevitch, 2007; Boukens et al., 2009). Intrinsic heterogeneity within normal hearts contributes to the development of arrhythmias under certain conditions (Bishop et al., 2013). Premature beats in the heterogeneous area that occurred during a certain time duration (i.e., VW) would lead to unidirectional conduction block and reentry arrhythmias. In most cases, the VW is rather small; however, it can be significantly enlarged in some pathological conditions due to augmented regional heterogeneity, such as ischemia (De Bakker et al., 1988; Yuuki et al., 2004) and heart failure (Roden, 2003; Coronel et al., 2013). For the local inflammatory condition, our simulation results suggested that the repolarization of the tissue developing inflammation was significantly delayed, which in turn contributed to an apparent electrical heterogeneity around the local area. Such regional dispersion of repolarization, as an arrhythmogenic substrate, will be further amplified in higher but physiologically relevant pacing frequencies and eventually lead to arrhythmia. Regarding interspecies differences, the simulation results suggested that inflammation weakened the repolarization ability of both rat and human myocytes, but there were obvious interspecies variances behind it, as repolarization is notably different in rodents compared to large mammals. In particular, the cytokine-induced APD prolongation in rat myocytes was mainly attributed to the decreased *I*_to_; in contrast, the APD prolongation in human ventricular cells resulted mainly from the reduced *I*_Kr_. It has been demonstrated that *I*_to_ is the major repolarization current in rat myocytes (Zhao et al., 2012), but for humans, *I*_to_ is only one of various repolarizing currents and is mainly involved in repolarizing phase 1 of cardiac AP. In comparison, hERG (the gene that codes for the alpha subunit of *I*_Kr_) is abundantly expressed in human ventricles, and *I*_Kr_ plays a critical role in repolarizing during cardiac AP (Ledford et al., 2022; Cheng and Kodama, 2004). This interspecies variation has also been widely observed in other studies. For instance, when used to treat carbon monoxide (CO)-induced arrhythmias, ranolazine was shown to be effective in inhibiting CO-induced EAD in rat cells (Dallas et al., 2012), but it exacerbated EADs and even caused oscillation in guinea pigs (which have wide APs similar to those in humans). In a recent study (Morotti et al., 2021), Morotti et al. examined the influences of interspecies differences on animal experimentation and drug efficacy assessment, and they created cross-species translators of electrophysiological responses to translate the drug-induced effects experimentally observed in myocytes from animal models to predict the effects that these perturbations would cause in humans. In summary, although our simulations demonstrated that inflammation caused similar reentry arrhythmias in rats and humans, the underlying mechanisms were different among various species and this needs to be taken into account, especially when translating experimental findings regarding drug efficacy and safety from animal models to clinical use.

### 4.2 Limitations

It should be noted that the combined effect of three cytokines was assumed to be an additive effect of each cytokine in the present work. Kumar et al. (Kumar et al., 1996) reported a synergistic inhibitory effect of TNF-α and IL-1β on ventricular contractility, where the concentration of each cytokine under conditions of a combination was much lower than those acting alone. Such synergistic effects and the potential antagonistic effects among cytokines were not considered in this study.

Although the present study followed the experimental setup in the original reports exactly, the concentrations of inflammatory cytokines used in previous experiments were generally higher than those observed under pathophysiological conditions. Specifically, Liu et al. reported a median IL-6 serum level of 4809 pg/ml in a nonsurvivor group of patients with sepsis (Liu et al., 2021). Liu et al. reported that the pathophysiological level of TNF-alpha was approximately 0.15 ng/ml in the serum of heart failure patients (Liu and Zhao, 1999). Monnerat et al. reported a serum IL-1β level of 60 pg/ml in diabetic mice (Monnerat et al., 2016). Therefore, our simulation results reflected an extreme condition of inflammation (i.e., sepsis). To account for this, we have considered another ‘mild’ inflammatory condition by halving the concentrations of the inflammatory cytokines; however, caution should still be taken when translating these findings to clinical use.

The effects of inflammatory cytokines on ion channels were assumed to be consistent by setting specific constant change ratios to the conductance of the involved ion channels. However, inflammation is a dynamic process with complicated mechanisms. For example, there have been studies showing that inflammatory cytokines could increase sympathetic activity to inhibit cytokine production; however, it also deserves to be noted that hyperactive sympathetic nerves could directly influence the function of ion channels by phosphorylation in myocardial cells and may induce arrhythmias (Lazzerini et al., 2017). The above dynamic process, as a kind of negative feedback mechanism, could change the concentrations of inflammatory cytokines in serum, which was not incorporated in our simulations. In addition, inflammatory cytokines could also affect the electrophysiological function via complicated indirect pathways in addition to the introduced direct modulations of ion channels and calcium handling. These indirect pathways include, but are not limited to, the following two aspects: 1) inflammatory cytokines could cause chronic remodeling and myocardial fibrosis, thus increasing the susceptibility of cardiac tissue to arrhythmias in chronic heart failure patients (Dick and Epelman, 2016); 2) current evidence suggests that TNF-α may promote the formation of atherosclerotic plaques by upregulating the expression of multiple protein molecules (e.g., adhesion molecule-1) in the vascular wall (Ohta et al., 2005), which would exacerbate the ischemic condition of hearts and lead to ischemic-related arrhythmias. As we mentioned above, inflammation has complex mechanisms and effects that require further simulation studies. Above all, these indirect pathways play critical roles in inflammation-mediated arrhythmias but they were not investigated in this study.

Limited to the sources of the experimental data, the proarrhythmic mechanisms investigated in this study focused mainly on ventricular arrhythmia. In fact, accumulating evidence has shown that there is a strong link between inflammation and postoperative atrial fibrillation. Maesen et al. reported an overlapping time course of atrial fibrillation occurrence after cardiac surgery and the activation of the complement system with the release of proinflammatory cytokines, suggesting potential roles of inflammation in triggering postoperative atrial fibrillation (POAF) (Maesen et al., 2012). Heijman et al. observed that postoperative inflammation along with preexisting Ca^2+^-handling abnormalities contributed to the formation of DADs and thus led to POAF (Heijman et al., 2020). The above postoperative atrial fibrillation, as a widely accepted form of inflammation-induced arrhythmia, warrants further research.

## 5 Conclusion

In this study, we conducted an in silico investigation using a multiscale virtual heart to explore the proarrhythmic mechanisms of inflammation. Inflammatory cytokines directly affect the function of ion channels and thus cause prolongation of AP and augmentation of transmural dispersion. The augmentation of the transmural dispersion would increase the vulnerability to arrhythmia (e.g., the greater VWs). In addition, the prolongation of AP contributes to significant pathological heterogeneity and provides extra substrates for inducing arrhythmia under conditions of local inflammation. In the case of global inflammation, the QT interval and the minimum PCL for normal 1:1 conduction are both enhanced, indicating a greater proarrhythmic effect. In summary, the present study provides new insights into the underlying mechanisms of the systemic inflammatory response to arrhythmia.

## Data Availability

The original contributions presented in the study are included in the article/Supplementary Material, further inquiries can be directed to the corresponding author.

## Abbreviations

VF, ventricular fibrillation; TNF-α, tumor necrosis factor-α; IL-1β, interleukin-1β; IL-6, interleukin-6; CVDs, cardiovascular diseases; COVID-19, coronavirus disease 2019; ECG, electrocardiogram; BCL, basic cycle length; AP, action potential; APD, action potential duration; ENDO, endocardial; EPI, epicardial; MID, middle; SR, sarcoplasmic reticulum; CV, conduction velocity; VW, vulnerable window; PCL, pacing cycle length; SERCA, sarco/endoplasmic reticulum Ca^2+^-ATPase; EAD, early-afterdepolarizations; DAD, delayed afterdepolarizations; POAF, post-operative atrial fibrillation; TdP, torsade de pointes; TRIaD, triangulation, reverse use dependence, instability and dispersion.
