# Supplementary material for: Mechanistic Insights Into Inflammation-Induced Arrhythmias: A Simulation Study
Source: Front Physiol. 2022 May 30;13:843292. doi: 10.3389/fphys.2022.843292 (PMC9196871; doi:10.3389/fphys.2022.843292)
Supplement: Supplementary file 3 [file DataSheet1.DOC]

Supplementary Material

# Supplementary Data

The source codes of this study could be downloaded can be downloaded via the link: https://github.com/810659784/Inflammation-induced-arrhythmia.git

# Supplementary Figures


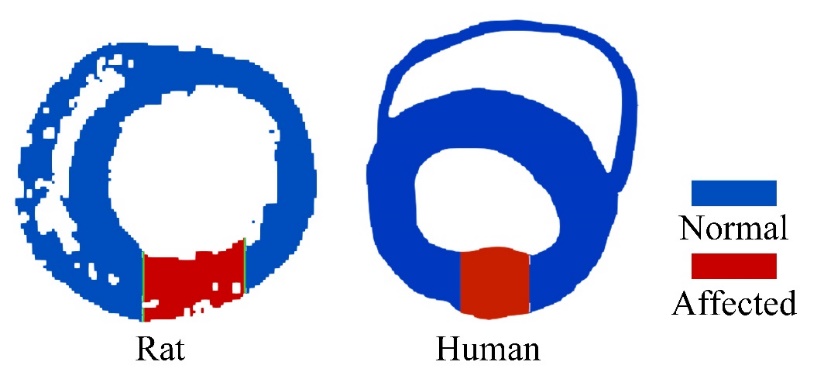


**Figure S1.** The schematic diagram of the local inflammatory condition. The inflammatory tissue area

is highlighted in red.


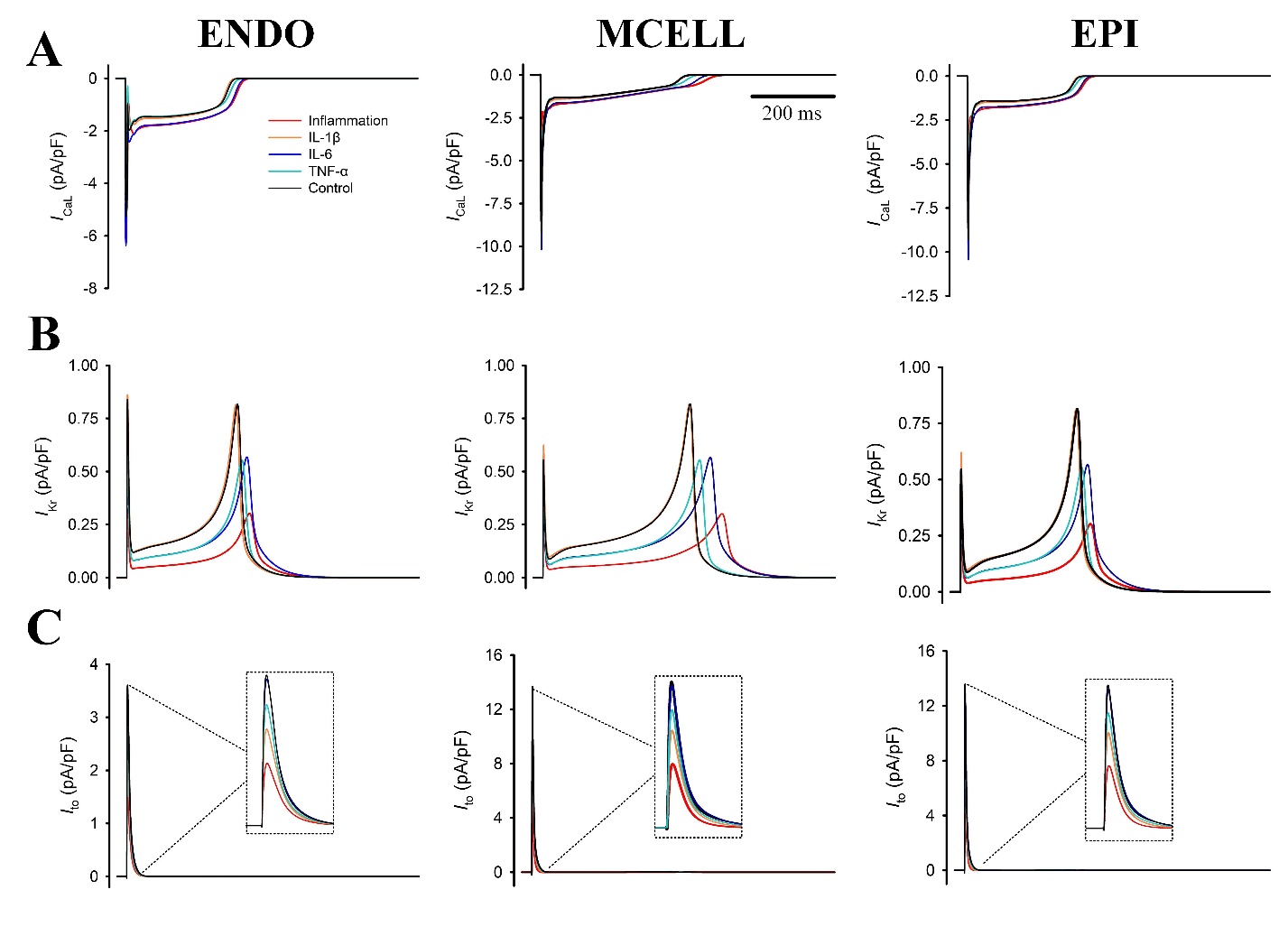


**Figure S2**: Current traces under different cytokines in human ventricular cell models. (A) Corresponding current profiles for *I*_CaL_ (B) Corresponding current profiles for *I*_Kr_ (C) Corresponding current profiles for *I*_to_.


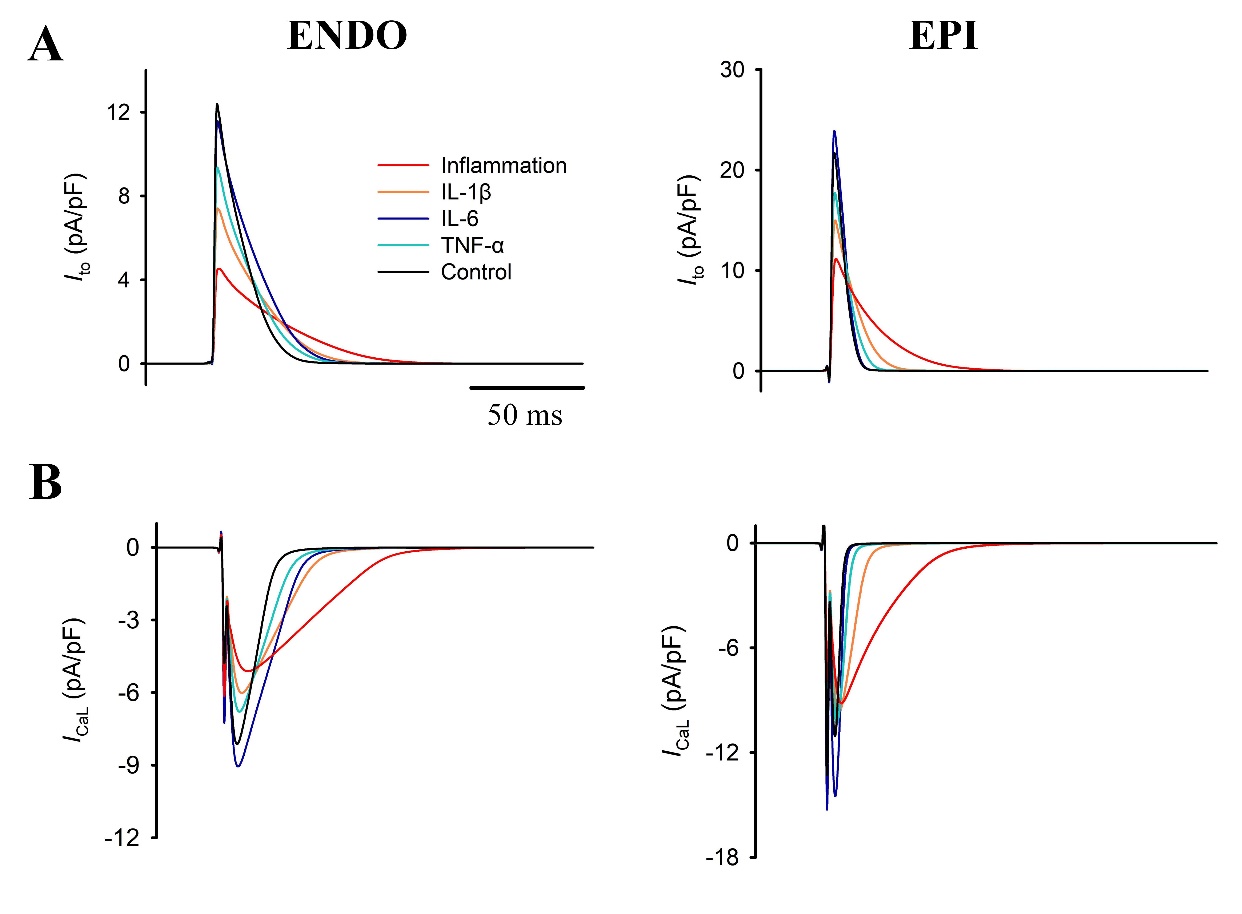


**Figure S3**: Current traces under different cytokines in rat ventricular cell models. (A) Corresponding current profiles for *I*_to_ (B) Corresponding current profiles for *I*_CaL_.


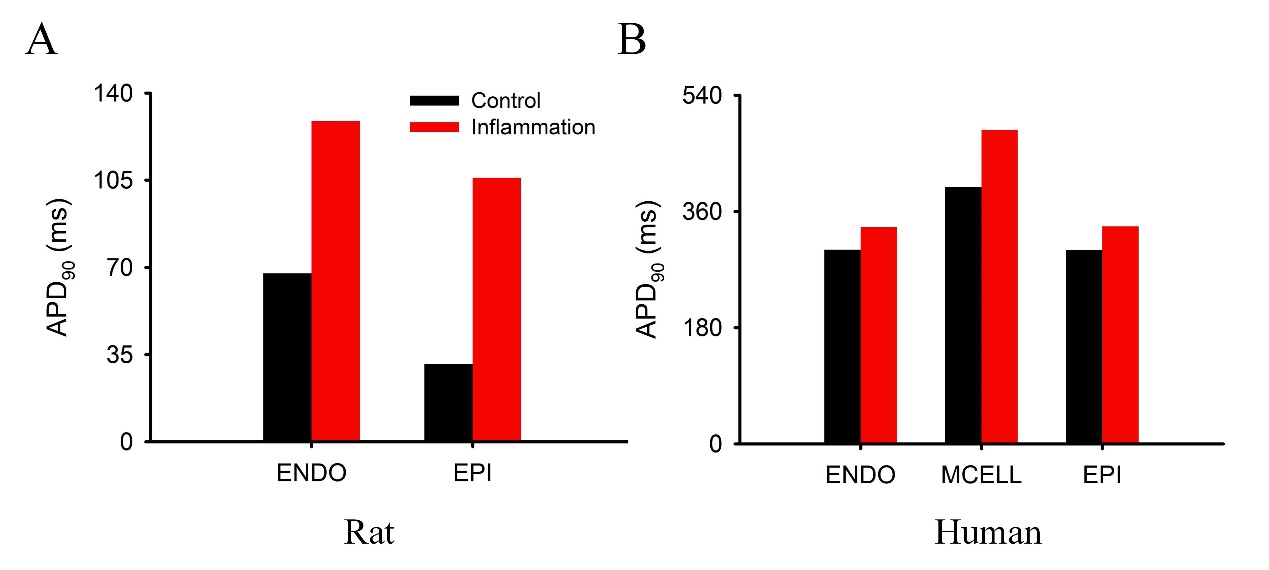


**Figure S4.** The comparison of APD_90_ between physiology and inflammation in two ventricular models, (A) for rat and (B) for human.


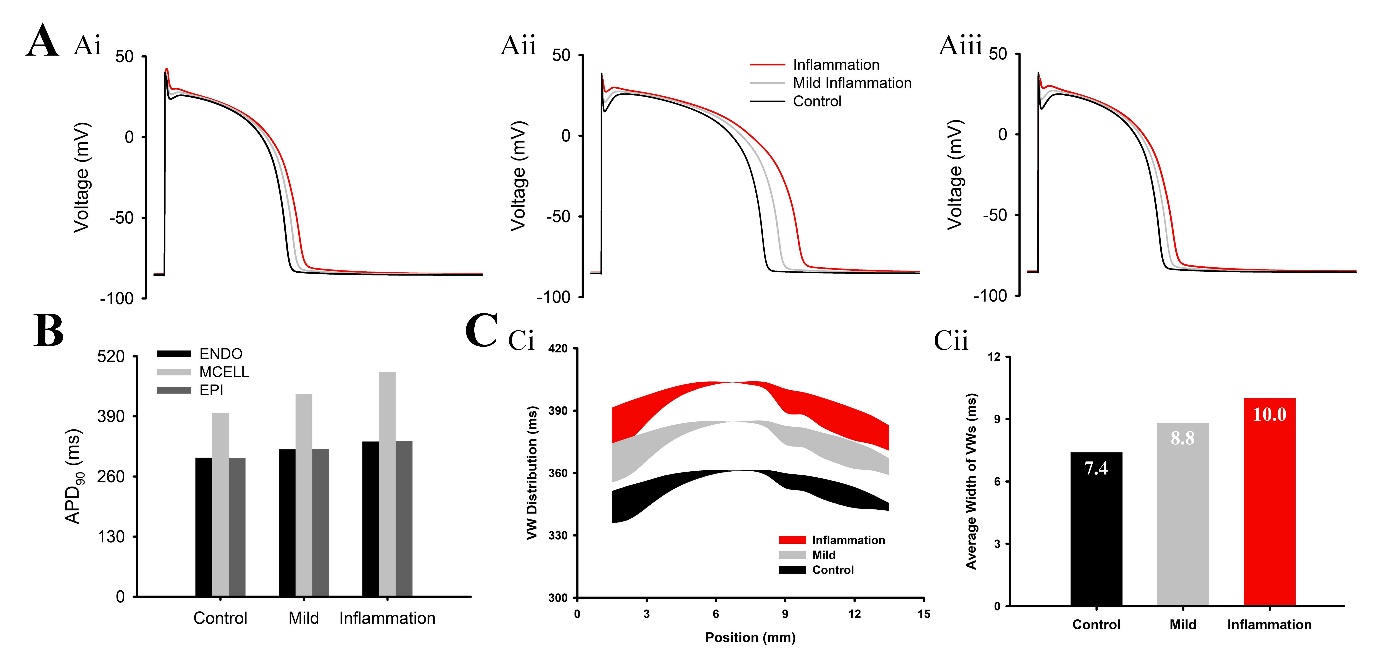


**Figure S5**. Simulation results under different levels of inflammation. (A) AP configurations of endocardial (Ai), Midcardial (Aii) and epicardial (Aiii) cells in different levels of inflammation. (B) The APD_90_ of three types of cells under normal and inflammatory conditions. (C) Measurement results of the vulnerable window in the 1-D strand model, (Ci) for VW distribution and (Cii) for average width of VWs. Note: the “Inflammation” in legend represents the effect of the original data in Table 1 and the “Mild” denotes the effect of the halving the original data.


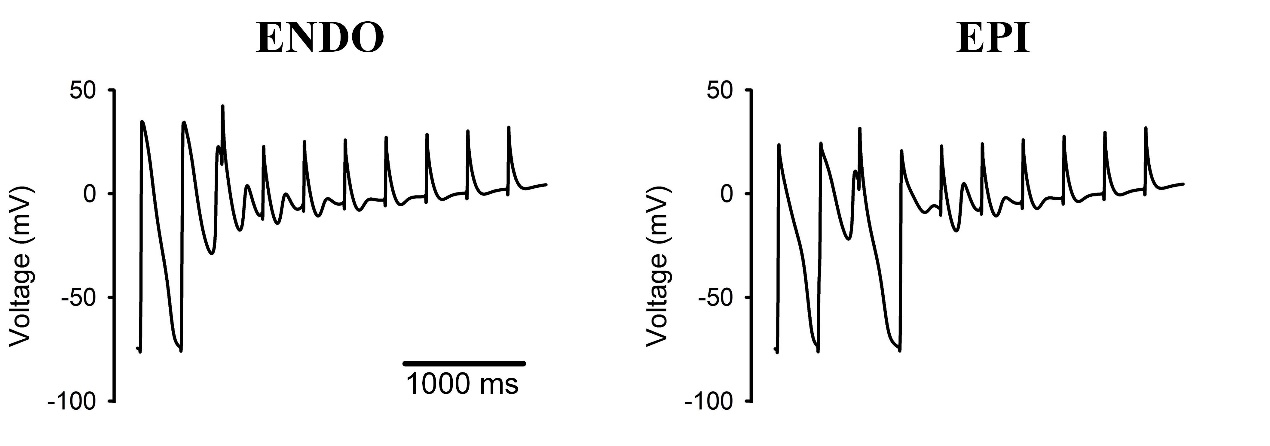


**Figure S6.** Potential model-dependency of the atypical phenomenon of complete repolarization failure. Above two figures showed simulated action potentials at a high stimulating frequency under inflammatory conditions, where both of the two cell types completely failed to repolarize. Such phenomenon was neither observed in human cells nor in control rat cells.
